# Colostrum avoidance practice and associated factors among mothers of infants less than six months in Chencha District: cross-sectional study

**DOI:** 10.1186/s40795-023-00674-4

**Published:** 2023-01-23

**Authors:** TekleBuche Asaro, Befikadu Tariku Gutema, Haymanot Nigussie Weldehawaryat

**Affiliations:** 1 Health Office of Bursa District, Sidama, Ethiopia; 2 School of Public Health, Arba Minch University, Arba Minch, Ethiopia

**Keywords:** Breastfeeding, Colostrum, Colostrum avoidance, Mothers, Cultural, Belief, Ethiopia

## Abstract

**Background:**

Colostrum avoidance is failure to feed first breast milk to a newborn baby for the first 2 to 3 days after delivery. The problem of avoiding colostrum is prevalent in Ethiopia. But it is not adequately addressed yet. Therefore, the purpose of this study was to assess prevalence of colostrum avoidance practices and associated factors among mothers of infants aged less than six months; and to explore barriers for colostrum feeding in ChenchaZuria District.

**Methods:**

A community-based cross-sectional study supplemented with a qualitative study was conducted in August 2020. The quantitative data were collected from 674 mothers selected by systematic sampling using a structured questionnaire. Both bi-variable and multi-variable binary logistic regression analysis was used to identify factors associated with the colostrum avoidance practices. The statistical significance was declared at a *p*-value < 0.05. The qualitative data were collected using in-depth interviews from breastfeeding mothers and thematic analysis was done manually.

**Result:**

The prevalence of the colostrum avoidance practice was 15.3% (95% CI: 11.4%- 18.2%). Late initiation of breastfeeding (AOR 4.15 95% CI 2.51–6.84), giving pre-lacteal feeding (AOR 3.16 95% CI 1.93–5.15), not using of postnatal care (PNC) service (AOR 1.79 95% CI 1.05–3.04), and having poor maternal knowledge regarding colostrum. (AOR 1.88 95% CI 1.14–3.08) were factors significantly associated with the colostrum avoidance practices. And in the qualitative part, cultural beliefs and misconceptions, community influence, and complementary feeding practices were found to be facilitators for the colostrum avoidance.

**Conclusion:**

About one in seven mothers practiced colostrum avoidance. Factors that contributed to the colostrum avoidance practices were breastfeeding initiation, pre-lacteal feeding, PNC utilization, and maternal knowledge regarding colostrum. Thus, efforts to prevent colostrum avoidance practices should focus on strengthening and promoting PNC services utilization, timely initiation of breastfeeding, and improving awareness creation activities on the importance of colostrum feeding and risks of pre-lacteal feeding.

## Introduction

Breastfeeding is a natural, inexpensive and an important for the growth and development of newborn babies [1]. The World Health Organization (WHO) and United Nation Children’s Emergency Fund (UNICEF) recommends that breastfeeding should be initiated within the first hours of life, provide colostrum from birth, provide exclusive breastfeeding for children up to six months of age, start appropriate complementary foods at six months, and continued breastfeeding until 24 months or beyond [2, 3].

Colostrum is the first thick yellowish breast milk, which is produced for the first 2 to 3 days after delivery. It is also distinct in volume, appearance, and composition compared to mature milk [4]. Colostrum acts as the first immunization a child receives and protects them from malnutrition [5–7]. Furthermore, colostrum can influence cell growth, differentiation, and function. And It can act as a growth promoter [8].

Colostrum avoidance is a failure to feed the first, thick and yellowish milk to the newborn baby. The practice includes squeeze out and throwing, pumping and discarding [9]. Even though WHO and UNICEF recommended initiating colostrum feeding within the first hour of birth, a higher number of mothers were avoiding feeding it to their infants [9]. As various studies reported, colostrum avoidance among mothers of newborn were documented in different parts of the world [10–12]. In sub-Saharan Africa, there is substantial variation in the magnitude [12–14]. In Ethiopia, the prevalence of colostrum avoidance ranges from 6.3% to 52.9% [15–20]. In many developing countries, women discard colostrum based on traditional or cultural beliefs by perceiving it as sour and difficult to digest, and harmful to the infant’s health. Therefore, they replace it with pre-lacteal feedings such as plain water, honey, formula, or animal milk [21–24]. In addition to the cultural beliefs different maternal related factors like lack of awareness and comorbidity, newborn related factors such as the need of special care due to the babies health condition and health care related factors are the barriers for colostrum feeding [25].

In Ethiopia, following international recommendations, the Federal Ministry of Health (FMoH) has established the National Nutrition Programs and National Infant and Young Child Feeding (IYCF) guidelines to promote breastfeeding practice including colostrum feeding of newborns [26]. And the colostrum avoidance practices were documented in many parts of country even after the implementation of these programs and guidelines [15, 17, 19, 20, 27]. However, most of these previous researches consider mothers having children aged less than 24 months as a study participant, which were highly prone to recall bias. Therefore, by using “mothers having infants aged less than six months” as a study participant and adding qualitative study this study assesses prevalence of colostrum avoidance practices and associated factors among mothers of infants aged less than six months; and explore barriers for colostrum feeding in Chencha Zuria District.

## Methods and materials

### Study area and period

The study was conducted in Chencha Zuria district, Southern Ethiopia. Chencha Zuria district is located about 511 km Southwest of Addis Ababa, the capital city of Ethiopia. Based on the district health office report, the total population of the district was 91,677 and it has 18,710 households with 3,172 live births. It is divided administratively into 34 kebeles (the lowest administrative unit in Ethiopia). In addition, it has 5 health centers and 34 health posts to provide MNCH services to the population. And the study was conducted from August 1 to 30, 2020.

### Study design

A quantitative community-based cross-sectional study supplemented by qualitative method was used.

### Population

Mothers having infants aged less than six months were the source population. Those mothers who gave birth to an infant aged less than six months and lived in the study area for at least 6 months were included in the study. However, mothers who were seriously ill or unable to give the required information during the data collection period were excluded from the study.

For the qualitative part of the study, the study participants were breastfeeding mothers who have infants aged less than 12 months.

### Sample size determination

The sample size was determined by using Epi info with the following assumptions: 95% confidence interval for a two-sided test; 80% of power; ratio of unexposed to exposed of 1:1; the proportion of outcome among mothers with timely initiation of breastfeeding (proportion of outcome in unexposed group) = 8.9%, and Adjusted Odds ratio of 2.4 [20]. Accordingly, the calculated sample size was 408 mothers. Adding 10% for non-response then multiplying by design-effect of 1.5the final sample size for this study was 674 study participants.

For the qualitative part, the data saturation approach was used to determine the sample size. Accordingly, eleven mothers were recruited for the interview.

### Sampling procedure

From the total 34 kebeles found in ChenchaZuria district, eleven kebeles were selected by lottery method. The study subjects were proportionally allocated to each kebele based on the number of women who gave birth in the respective kebeles. Then study participants were identified by systematic sampling method using the Health Extension Workers (HEWs) birth registry book as a sampling frame. After determining the starting mother using the lottery method, every other mother (K = 2) was selected as a study participant from the registration book list.

For the qualitative part of the study, the purposive sampling technique was used to recruit the study participants. Then eleven breastfeeding mothers who have infants aged less than 12 months were recruited for In Depth Interview (IDI).

#### Operational definitions

Colostrum avoidance is defined as a failure to feed the first, thick, and yellowish milk to a newborn baby that is produced in the first 2–3 days after delivery. It includes squeezing and throwing out or pumping and discarding [9].

##### Maternal knowledge about importance of colostrum feeding

Of the five questions relating to maternal knowledge the mothers who correctly answered three or more questions were considered as having good knowledge. And the mothers who correctly answered less than three questions were considered as having poor knowledge [23].

Pre-lacteal feeding is giving a liquid or foods other than breast milk prior to the establishment of regular breastfeeding with in the first three days of life of new born infant.

Timely initiation of breastfeeding: If an infant put on mother’s breast to feed within one hour (including one hour) of birth.

### Data collection instrument and procedures

The quantitative data were collected using a structured and interviewer-administered questionnaire, which was developed from previous literature by adapting and modifying contextually to fit the local situation and research objectives [15, 17, 19, 20, 28]. The questionnaire was initially prepared in English then translated to Gamo language. Then to check its consistency, language experts again back translate it to the English language. Six diploma nurses collected the data via Open Data Kit (ODK) application using smartphones.

The qualitative data were collected by using a semi-structured interview guide by the IDI technique. Trained BSc Nurse with the assistance of two note-takers collected the data. And they recorded the interviews via digital voice recorder.

### Data quality control

Pre-testing of the quantitative questionnaire was carried out on 5% of the sample size before starting the actual data collection to improve the clarity and understandability of the tool. The data collectors trained for two days collected the data. The investigators supervised and coordinated the overall activity of the data collection. The investigators also reviewed and checked the collected data daily for completeness and consistency.

To assure the data quality for qualitative part data was triangulated in space by gathering information from various locations inside the study area. Additionally, in order to triangulate the data in person variety of study participants with various socio-demographic traits were selected. These ideas are now included in the recent submission of the manuscript.

### Data processing and analysis

The quantitative data from the ODK briefcase were exported to SPSS version 25 statistical package for the further analysis. Frequencies, proportions, mean, standard deviation, tables, and graphs were used to describe the data. Principal Component Analysis (PCA) were applied to analyze wealth status of the study participants. Binary logistic regression analysis was used to identify associations between dependent and independent variables. In the bi-variable logistic regression analysis, variables which were statistically significant at *p*-value < 0.25 and biologically plausible were candidate variable for multi-variable logistic regression analysis. Confounders were controlled by running step-wise backward logistic regression analysis. Multi-collinearity among independent variables was checked by using Variance Inflation Factor (VIF) and the values of all variables found within 1.0 and 2.0. The fitness of logistic model was assessed by using the Hosmer and Lemeshow Goodness of fit test with *p*-value of 0.776.The degree of association of independent variables with the dependent variable was assessed using AOR with 95% confidence interval and *p*-value < 0.05 in the final multi-variable model.

The qualitative data was analyzed manually by using thematic analysis approach. Qualitative data transcribed word by word, translated into English language and coded. Then, coded data grouped into the three key themes guided by the literature and an interview guide.

## Results

### Socio demographic characteristics of study participants

Six hundred forty-seven mothers participated in the study with a response rate of 96.0%. The mean (± SD) age of the study participants was 29.2 (± 6.02) years. The majority of the respondents were married (93.4%) and from the Gamo ethnicity group (93.8%). Regarding to educational status, 42.5% of the participants had attended formal education. Most of the participants (79.4%) were housewives (Table 1).

**Table 1:** Socio-demographic characteristics of mothers having infants less than 6 months of age in ChenchaZuriaDistrict, Southern Ethiopia,2020

Variable	Categories	Frequency	Percent
**Age group**	18–27	274	42.3
	28–37	302	46.7
	38–47	71	11.0
**Religion**	Orthodox	320	49.5
	Protestant	307	47.4
	Others^**a**^	20	3.1
**Ethnicity**	Gamo	607	93.8
	Zeysie	17	2.6
	Others^**b**^	23	3.6
**Occupational status of mother**	Housewife	514	79.4
	Merchant	53	8.2
	Governmental employee	24	3.7
	Student	20	3.1
	pit trading and daily laborer	36	5.6
**Educational status of mother**	Formal education	275	42.5
	Able to read and write	208	32.1
	Unable to read and write	164	25.3
**Marital status of the mothers**	Married	604	93.4
	Single/Divorced	29	4.4
	Widowed	14	2.2
**Occupational status of husband (*N* = 604)**	Farmer	405	67.1
	Merchant	64	10.6
	Daily laborer	55	9.1
	Governmental employee	52	8.6
	Others	28	4.6
**Educational status of husband (*N* = 604)**	Formal education	270	44.7
	Able to read and write	202	33.4
	Unable to read and write	132	21.9
**Household head**	Father	555	85.8
	Mother	44	6.8
	Grandfather	39	6.0
	Grandmother	9	1.4
**Wealth quintile**	Lowest	130	20.1
	Second	107	16.5
	Middle	150	23.2
	Fourth	143	22.1
	Highest	117	18.1

^**a**^Other include Adventist, Muslim; ^**b**^Other include Wolayta,Goffa, and Amhara

### Health care service utilization and obstetric history of the mothers

Most mothers (72.2%) received AntenatalCare (ANC) during their period of last pregnancy. During ANC follow-up visits, 63.4% of mothers were counseled about breastfeeding. From the participants, 27.4% gave birth at home and the rest got assistance from traditional birth attendants, neighbors, and friends. Similar to ANC follow-up, 72% of the mothers had postnatal care (PNC). Out of the total participant women, 81.8% were multiparous and the majority (85.8%) gave birth by normal delivery (Table 2).

**Table 2:** Health care service utilizationand obstetric history of the mothers having infant less than 6 months of age,at ChenchaZuria District, 2020

Variables	Categories	Frequency	Percentage (%)
**Attendance of ANC**	No ANC visits	180	27.8
	1–3 ANC visits	314	48.5
	4 -8ANC visits	153	23.6
**Participation in PWF**	Yes	365	56.4
	No	282	43.6
**Place of delivery**	Health facility	470	72.6
	Home	177	27.4
**PNC service utilization**	Yes	470	72.6
	No	177	27.4
**Number of parities**	Multi-Para	529	81.8
	Prim-Para	118	18.2
**Mode of delivery**	Normal delivery	555	85.8
	Instrumental delivery	48	7.4
	C/S delivery	44	6.8

### Breastfeeding practices and infant characteristics

Nearly half of the infant were males and 59.2% were a birth order of 2–3. The study showed that 79.6% of the mothers initiated breastfeeding within 1 h of birth. The most commonly mentioned reasons for delayed breast feeding were delayed breast milk secretion (37.9%) and Infant illness (35.6%). Maternal illness (13.6%), Delivery with cesarean Sect. (9.1%) and breast diseases (3.8%) were mentioned as a reason for delayed breast feeding. Regarding pre-lacteal feeding, 27.2% reported that they gave pre-lacteal feeds to their infants within the first 2 to 3 days of birth. The types of pre-lacteal foods given include butter (43.2%), pain water (40.3%) and caw milk (16.5%).

Of the mothers included in this study 15.3% (95% CI: 11.4%—18.2%) avoided colostrum. The most commonly reported reasons for colostrum avoidance were: that assuming colostrum feeding is not good for a newborn baby (41.4%) and assuming colostrum feeding causes abdominal discomfort & diarrhea (29.3%).Infant inability to feed (18.2%), tradition (7%) and the color (4%) were also mentioned as a reason to avoid colostrum. Grandparents (37.4%), traditional birth attendants (29.3%), friends (20.2%), and husband (3%) are the persons who advise the mothers to avoid colostrum. Only 10.1% of the mothers avoid colostrum by their own decision **(**Table 3).

**Table 3:** Breastfeeding practices related characteristics of study participant of the mothers having infant less than 6 months of age, at ChenchaZuria District, 2020

Variables	Categories	Frequency	Percent (%)
**Sex of the infant**	Female	338	52.2
	Male	309	47.8
**Birth order of the infant**	Birth order one	138	21.3
	Birth order 2–3	383	59.2
	Birth order 4 and more	126	19.5
**Breast feeding initiation time**	Within 1 h of birth	515	79.6
	1–11 h after birth	98	15.1
	12 –23 h after birth	32	4.9
	After 24 h of birth	2	0.3
**Pre-lacteal feeding**	No	471	72.8
	Yes	176	27.2
**Colostrum avoidance**	No	548	84.7
	Yes	99	15.3
**Maternal knowledge about importance of colostrum feeding**	Poor knowledge	225	34.8
	Good knowledge	422	65.2

#### Factors associated with colostrum avoidance practice

In the bi-variable analysis, occupational status of the husband, household head, birth order, participating in pregnant women forum, place of birth, breastfeeding initiation time, pre-lacteal feeding, PNC utilization, and maternal knowledge about the colostrum were candidate variables for multivariable logistic regression analysis.

Mothers who initiated breastfeeding after one hour of delivery were nearly four times (AOR 4.15, 95% CI: 2.51–6.84) more likely to discard colostrum compared to those who initiated breastfeeding within one hour after delivery. Mothers who had been given pre-lacteal feeding were nearly three times (AOR3.16, 95% CI: 1.93–5.15) more likely to discard colostrum as compared to those who did not practice pre-lacteal feeding. The odds of discarding colostrum among mothers who did not attend PNC services were 1.79 times (95% CI: 1.05–3.04)higher compared to those who did not attend PNC services. The likelihood of discarding colostrum was higher (AOR 1.88, 95%CI: 1.14–3.08) among mothers who had poor knowledge about the importance of colostrum feeding compared to their counterparts (Table 4).

**Table 4:** Bi-variable and multi-variable binary logistic regression analysis of factors associated with colostrum avoidance practices of the mothers having infant less than 6 months of age, atChenchaZuria District, 2020

Variable	Categories	Colostrum avoidance		COR (95% CI)	AOR (95% CI)
		Yes	No		
		N(%)	N(%)		
**Household head**	Father	88(15.9%)	467(84.1%)	1	1
	Mother	7(15.9%)	37(84.1%)	1.00(0.43–2.31)	1.38(0.53–3.59)
	Others	4(8.3%)	44(91.7%)	2.07(0.73–5.92)	1.09(0.32–3.74)
**Occupation of husband**	Farmer	74(18.0%)	337(82.0%)	1	1
	Merchant	10(14.1%)	61(85.9%)	2.01(0.84–4.85)	1.88(0.71–3.19)
	Daily laborer	6(9.8%)	55(90.2%)	2.32(1.12–4.80)*	1.98(0.84–3.19)
	Others	9(8.7%)	95(91.3%)	2.32(1.12–4.80)*	1.98 (0.84–4.66)
**Breast feeding initiation**	Late initiation	51(38.6%)	81(61.4%)	6.13 (3.87–9.70)*	4.15(2.51–6.84)*
	Timely initiation	48(9.3%)	467(90.7%)	1	1
**Pre-lacteal feeding**	Yes	57(32.4%)	119(67.6%)	4.89(3.13–7.65)*	3.16(1.93–5.15)*
	No	42(8.9%)	429(91.1%)	1	1
**Participating in PWF**	No	53(18.8%)	229(81.2%)	1.61(1.04–2.47)*	0.69(0.39–1.20)
	Yes	46(12.6%)	319(87.4%)		
**Place of delivery**	Home	47(26.6%)	130(73.4%)	2.91(1.87–4.51)*	1.69(0.98–2.90)
	Health facility	52(11.1%)	418(88.9%)	1	1
**PNC utilization**	No	46(26.0%)	131(74.0%)	2.76(1.78–4.29)*	1.79 (1.05–3.04)*
	Yes	53(11.3%)	417(88.7%)	1	1
**Maternal knowledge about colostrum**	Poor knowledge	55(24.4%)	170(75.6%)	2.78(1.80–4.30)*	1.88(1.14–3.08)*
	Good knowledge	44(10.4%)	378(89.6%)	1	1
**Birth order**	Birth order one	26(18.8%)	112(81.2%)	1.28(0.77–2.12)	0.45(0.20–1.01)
	Birth order 2–3	59(15.4%)	324(84.6%)	1.86(0.92–3.74)	0.66(0.33–1.33)
	Birth order 4 & more	14(11.1%)	112(88.9%)	1	1

^*^statistically significant variable at *p* < 0.05 in multi-variable logistic regression analysis

##### Facilitators for colostrum avoidance

Eleven mothers participated in IDI and a mean (± SD) age of the study participants was 28.18(± 7.534) years, ranging from 20 to 42 years.The facilitators for colostrum avoidance were described in the three themes. These are cultural beliefs and misconceptions, community influence, and complementary feeding practices.

##### Cultural Beliefs and Misconception:

Mothers mentioned that the reasons for colostrum avoidance were perception related to colostrum feeding as not good for newborn babies and the feeding it causes abdominal discomfort and diarrhea. A 39 year old mother stated, "*… feeding colostrum to a newborn baby causes abdominal discomfort and diarrhea, and we discard it for first two days”* (Participant 6*)*.A 42-year-old mother also said, *“… feeding colostrum to a newborn was believed unsafe in the old days, but now as educated by HEW, it is used as foods for newborn baby, and it prevents against illness…and we have fed it to our babies”* (Participant -3).

##### Community influence:

The mothers mentioned that grandmothers, traditional birth attendants, and elders were the influential persons for the colostrum avoidances. They thought that colostrum is not good for the newborn baby. Therefore, it should not be given for the first 2 to 3 days. A 24 year mother stated, *“… grandmothers and elders in the family told us first breast milk is not good for an infant so, we did not offer it to the newborn baby until second days of birth”* (Participant-10). A 23-year-old mother said, *“… we did not feed colostrum to our baby for first two days of birth as instructions given by traditional birth attendants and grandmothers”* (Participant -6).

##### Pre-lacteal feeding:

Mothers mentioned that since, they perceive colostrum as harmful for the babies' health; they give butter, cow milk, or water for the first two to three days. A 39-year-old mother said, “*… instead of the colostrum we give cow’s milk and water till regular milk secretion starts”* (Participant- 6).

## Discussion

The result of this study shows that The prevalence of colostrum avoidance practice among mother having infants aged less than six months in Chencha Zuria district was 15.1% (95% CI: 11.4%- 18.2%). And This study reveals that, a breast feeding initiation time, pre-lacteal feeding, PNC service utilization and maternal knowledge about the importance of colostrum feeding had statistically significant association with colostrum avoidance practice.

The findings of this study shows that the prevalence of colostrum avoidance practice among mothers who had infants less than 6 months of age was 15.3%. This result is comparable with previous studies conducted in Kombolcha Town, Raya Kobo, Bahir Dar city that ranges from 11.4% to 16.1% [15, 20, 29]. Studies conducted in Pakistan (27.9%), Nepal (64.39%), and South Sudan (38.8%)showed significanlty higher prevalence of colostrum avoidance compared to this finding [11, 14, 30]. Similarly, studies conducted in Northern parts of Ethiopia in the Dabat (52.9%), Northwest Ethiopia(22.1%) and Motta town (20.3%), showed higher prevalence compared to the finding of this result [16, 27, 31]. The possible explanations for this this difference in prevalence might be the difference in socio cultural and health service settings with the mentioned studies.

In contrary, In comparison to this study, investigations from Tamil Nadu (India), Kenya, and Axum(Ethiopia) revealed lower prevalence of colostrum avoidance [19, 32, 33]. This variation could be due to difference in study sample size, health service settings, and difference in breastfeeding practice.

The quantitative result shows that the reasons for colostrum avoidance were mothers’ belief that colostrum feeding is not good for newborn baby; and it can cause abdominal discomfort and diarrhea. As the study participants reported, the most influential individuals for colostrum avoidance were grandmothers and traditional birth attendants. This finding was consistent with study conducted in Nepal, India, Pakistan, Kombolcha town and Raya Kobo [10, 11, 15, 20, 30].

Furthermore, Mothers who initiated breastfeeding after one hour of delivery were 3 times more likely to discard colostrum compared to mothers who initiated breastfeeding within one hour after delivery. This finding is in line with prior study conducted in in North Wollo and Raya kobo [17, 20]. This may be explained by the fact that mothers who discarded the colostrum might take more time to discard it and initiate breastfeeding later [20]. It might also be due to the fact that mothers who tend to initiate breastfeeding later has a chance to be far from the eyes of the health professionals so that they will have room to discard colostrum.

This study also reveals that Mothers who practiced pre-lacteal foods were more likely to avoid colostrum compared to those mothers who did not give pre-lacteal foods. This is consistent with findings of previous studies in North Wollo Zone [16]. This may be due to their belief that pre-lacteal foods have importance for infant’s health. Additionally, mothers believe that giving colostrum to a newborn baby would cause diarrhea and stomach pain [25].

Furthermore, maternal knowledge about the importance of colostrum feeding was statistically associated with the colostrum avoidance practice. This finding is consistent with the studies conducted in rural Bangladesh, Axum town, Raya Kobo and North Wollo [17, 19, 20, 34]. On the other hand, PNC service utilization had a significant association with colostrum avoidance practices. This finding was consistent with study conducted in Axum town [19]. This might be due to the fact that mothers who attend the PNC services during might have access to the counseling sessions by health care workers on the importance of the colostrum feeding, and thereby more likely to practice colostrum feeding to newborn.

The qualitative part of this study shows that, breast milk produced soon after delivery was perceived, as “it is not good for a newborn baby”. It was believed that colostrum cannot be digested by the infant’s fragile digestive system. Because colostrum was thought to be bad to the infant; butter, cow milk or water was given for the first two to three days after birth. This result is consistent with study conducted in Pakistan, Guatemala, Northwest Nigeria, North Wollo and Raya Kobo; Northern Ethiopia [17, 20, 35–37].

Mothers reported that barrier for not feedings of colostrum is the community influence. Grandmother, traditional birth attendants and elders encourage colostrum avoidance practices in the community and discourage feeding of it. Nevertheless, as some participants of the study reported, currently community view about colostrum feeding is now changing due to health education given by HEW and the majority of women were providing it to newborn.

The limitation of this study is social desirability bias because respondent may answer questions in a manner that would be viewed favorably by others. Additionally, as the study is conducted at a single point in time, it is impossible to determine the temporal relationship between the factors and the outcome.

## Conclusion

Nearly one from seven mothers among mothers of children aged less than six month practiced colostrum avoidance in study area. Breastfeeding initiation time, pre-lacteal feeding, PNC service utilization and maternal knowledge about the importance of colostrum feeding found to be the factors significantly associated with colostrum avoidance practices. As a result, policymakers should develop and implement effective guidelines to maximize PNC utilization, initiate breastfeeding at the appropriate time, and avoid pre-lacteal feeding, which were also identified as a facilitator in the qualitative study. Furthermore, the qualitative part shows that the culture-related beliefs and misconceptions among mothers and the community are barriers for colostrum feeding practices. Thus, policy makers should work to enhance maternal knowledge of colostrum and work to change the cultural beliefs and misinterpretations of the community.

## Data Availability

The datasets used and/or analyzed during the current study are available from the corresponding author on reasonable request.
